# Brain imaging in Kufs disease type B: case reports

**DOI:** 10.1186/s12883-015-0357-6

**Published:** 2015-07-04

**Authors:** Roberto Di Fabio, Claudio Colonnese, Filippo Maria Santorelli, Liliana Pestillo, Francesco Pierelli

**Affiliations:** Department of Medical-Surgical Sciences and Biotechnologies, Sapienza University of Rome, Corso della Repubblica 79, Latina, Italy; Department of Neurology and Psychiatry, Sapienza University of Rome, Rome, Italy; IRCCS Stella Maris, Pisa, Italy; ASL, Fondi, Italy; IRCCS Neuromed, Pozzilli, Italy

**Keywords:** Neuronal ceroid lipofuscinosis, Type B Kufs, Cathepsin F, *CTSF*, Brain MRI

## Abstract

**Background:**

The clinical traits of Kufs disease (KD) type B (CLN13), an adult-onset neuronal ceroid lipofuscinosis (NCL), are well established according to the neurological features of the cases reported with mutations in *CTSF*.

The neuroradiological characteristics of this uncommon disease have not yet been outlined.

**Case presentation:**

We hereby report the brain MRI features in two Caucasian women who carried homozygous mutations in *CTSF*, providing a short review of the neuroradiological findings of other common NCLs.

Together with a brain volume reduction, the two cases showed white matter hyperintensities and thinning of the corpus callosum at onset of the cognitive decline.

**Conclusion:**

White matter hyperintensities associated with volume reduction of the corpus callosum may be present at the beginning of the behavioural changes in CLN13 and represent further clues for searching mutations in *CTSF*.

## Background

Mutations in *CTSF*/CLN13, encoding cathepsin F, have recently been reported in Kufs disease (KD) type B (MIM 606725) [1, 2], an autosomal recessive, adult-onset, neuronal ceroid lipofuscinosis (NCL). The clinical features of this rare form of NCL are well established on the basis of findings in ten patients in four families and include dementia with motor disturbances, commonly associated with facial dyskinesias, in the absence of visual failure. The detection of fingerprint profiles or granular osmiophilic deposits by electron microscopy was mandatory for definitive diagnosis, although the significance of the biopsy in the diagnostic process of KD has been recently questioned [3], and then significantly reduced by the cloning of *CTSF*.

From a clinical standpoint, absence of visual impairment in young adults showing cognitive decline with a possible autosomal recessive inheritance should suggest CLN13. Although diffuse cerebral atrophy was detected in four cases belonging to three unrelated Caucasian families [1], the neuroradological features of such disease remain largely undescribed. The report of the neuroradiological changes and the time-frame in which these features are highlighted in additional patients with mutations in *CTSF* may represent a useful support for the clinician to disclose potential CLN13 both in sporadic and familial cases.

We report the results of brain MRI in two KD patients already described in full elsewhere [2].

## Case presentation

**Fig. 1:**
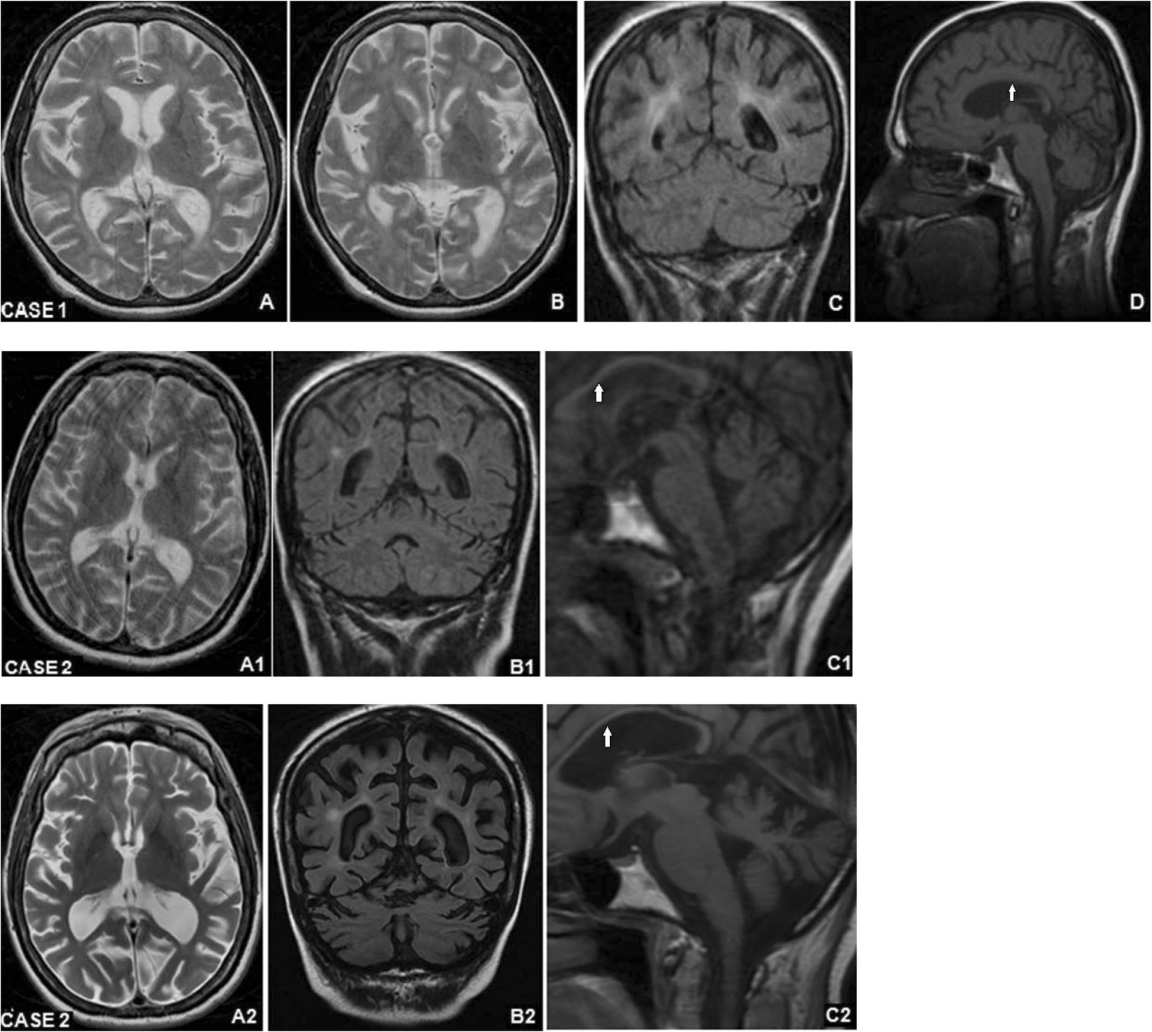
Brain MRI imaging in two patients with Kufs disease type B. *Case 1*. Axial T2-TSE (**a** and **b**), coronal T2-FLAIR (**c**), and sagittal T1-TSE (**d**) sections at onset of cognitive impairment showing cortico-subcortical atrophy, predominant in the parieto-occipital regions and the cerebellum, white matter hyperintensities and thinning of the corpus callosum (*arrow*). *Case 2*. Images at onset of cognitive impairment: axial T2-TSE (**a1**), coronal T2-FLAIR (**b1**) and sagittal T1-TSE (**c1**) sections displayed brain atrophy, mainly involving the cerebellum, periventricular hyperintensities and thinning of the corpus callosum (*arrow*). Follow up imaging: axial T2-TSE (**a2**) and coronal T2-FLAIR (**b2**) and sagittal T1-TSE (**c2**) scans taken in the same patient 9 years later

A 43-year-old woman (case 1) presented at the age of 23 years clusters of tonic-clonic seizures, fully controlled by a combination of sodium valproate (1250 mg/day) and carbamazepine (1200 mg/day). Her previous medical history was unremarkable except for several mild head traumas. No MRI scan were obtained at that time. From the age of 30, the patient showed rapid cognitive decline, postural tremor, sporadic myoclonic jerks in the lower limbs, and transient facial dyskinesias. Her vision was preserved. Brain MRI showed cerebral atrophy, mainly in the parieto-occipital regions, cerebellar atrophy and T_2_-weighted hyperintensities in the periventricular areas (Fig. 1).

A 45-year-old woman (case 2), a cousin of case 1, had experienced tonic-clonic seizures since the age of 21, controlled by sodium valproate (1000 mg/day), phenobarbital (100 mg/day) and zonesamide (200 mg/day). In her 30s, the patient developed cognitive impairment, ideomotor apraxia, cerebellar dysarthria and inappropriate laughter. Her sight was spared. Brain MRI, carried out when the neurological signs were noted, showed cortico-subcortical atrophy, periventricular white matter hyperintensities on T2-weighted scans, cerebellar atrophy, and thinning of the corpus callosum (Fig. 1).

Both patients belonged to the largest family with KD type B reported to date in which a homozygous c.213+1G>C mutation in CTSF was detected [2].

## Conclusion

We here reported the neuroradiological characteristics of two additional patients with KD type B in which, together with brain volume reduction, early periventricular and deep white matter hyperintensities and atrophy of corpus callosum were detected.

Careful neuroimaging in patients with suspected NCL may confirm the diagnosis, opening the way for genetic counselling and providing a basis for neurological screening in other family members. In this regard, a detailed report on the neuroradiological features of patients with this rare form of NCL could be crucial to disclose new potential disease carriers when clinical features emerge.

The cerebral and cerebellar volume loss seen on neuroimaging in our patients are not features pathognomonic of CLN13, having also been described in cases of adult-onset NCL due to mutations in *DNAJC5* [4], *CLN6* [5], *CLN5* [6] and in patients with mutations in *GRN* [7].

Ceroid, lipofuscin-like, is an autofluorescent pigment, resistant to lipid solvents, that accumulates in the lysosomes not as part of normal senescence [8]. Neuronal loss, astrocytic proliferation and macrophage infiltration may result in the macroscopic changes disclosed by neuroimaging in CLN13, as observed in other NCLs [9].

Symmetrical hyperintensities in the dentate nucleus and hypointense thalami [10, 11], a typical finding in palmitoyl protein thioesterase-1-related NCL, were not found in our KD patients. The periventricular high-signals on T2-weighted images as well as the narrowing of callosal commissure were also detected in classic infantile-onset NCLs [12], but are not commonly seen in adult-onset NCLs both in genetically-confirmed [4–7] and clinically diagnosed cases [13–15]. According to our findings, these MRI changes, occurring at the onset of the neurological syndrome, may represent clues for a diagnosis of CLN13, although a detailed study of neuroimaging in additional KD type B cases is warranted to support this assumption.

In summary, in familial cases with early, adult-onset, autosomal-recessive cognitive decline without visual failure, brain atrophy as well as periventricular white matter hyperintensities and thinning of the corpus callosum should alert clinicians to a possible diagnosis of CLN13.

## Consent

Written informed consent was obtained from the patient for publication of this Case report and any accompanying images. A copy of the written consent is available for review by the Editor of this journal.

## Abbreviations

KD: Kufs Disease
NCL: Neuronal ceroid lipofuscinosis
MRI: Magnetic Resonance Imaging
